# Deletions of the region 17p11-13 in advanced melanoma revealed by cytogenetic analysis and fluorescence in situ hybridization

**DOI:** 10.1038/sj.bjc.6690022

**Published:** 1999-09-24

**Authors:** I Okamoto, H Pirc-Danoewinata, J Ackermann, J Drach, H Schlagbauer Wadl, B Jansen, K Wolff, H Pehamberger, C Marosi

**Affiliations:** Department of Internal Medicine I, Division of Oncology, University of Vienna, Währinger Gürtel 18–20, Vienna, A-1090, Austria; Department of Dermatology, Division of General Dermatology, University of Vienna, Währinger Gürtel 18–20, Vienna, A-1090, Austria; Department of Clinical Pharmacology, University of Vienna, Währinger Gürtel 18–20, Vienna, A-1090, Austria

**Keywords:** melanoma, p53, deletion, cytogenetics, fluorescence in situ hybridization, immunohistochemistry

## Abstract

The significance of the p53 tumour-suppressor gene in the oncogenesis of a variety of malignant tumours has been demonstrated over recent years. However, the role of p53 in human malignant melanoma is still unclear. Therefore, we investigated melanoma metastases from 11 patients cytogenetically and with fluorescence in situ hybridization (FISH) after short-term culture, employing a p53 region-specific probe for 17p13.1 and a probe detecting the centromere of chromosome 17. Furthermore, paraffin-embedded tissue samples from nine of these patients were investigated immunohistochemically for expression of the p53 protein. Deletions of the short arm of chromosome 17 were seen in six melanomas in cytogenetic analysis. With FISH, three malignant melanomas had clones with only one p53-allele and an additional four malignant melanomas showed a reduced number of signals at the p53 tumour-suppressor gene locus compared with signals for the centromeric region of chromosome 17. This was confirmed by immunohistochemistry. Our results suggest that the 17p11–13 region is frequently deleted in malignant melanomas and that p53 or other genes located on this band might contribute to the malignant potential of advanced melanoma. © 1999 Cancer Research Campaign


*British Journal of Cancer* (1999) **79**(1), 131–137
© 1999 Cancer Research Campaign

### Deletions of the region 17p11–13 in advanced melanoma revealed by cytogenetic analysis and fluorescence in situ hybridization

**I Okamoto[1,2], H Pirc-Danoewinata[1], J Ackermann[1], J Drach[1], H Schlagbauer Wadl[3], B Jansen[2,3], K Wolff[2], H Pehamberger[2] and C Marosi[1]**

[1]Department of Internal Medicine I, Division of Oncology, [2]Department of Dermatology, Division of General Dermatology and [3]Department of Clinical Pharmacology, University of Vienna, General Hospital, Währinger Gürtel 18–20, A-1090 Vienna, Austria

**Summary** The significance of the p53 tumour-suppressor gene in the oncogenesis of a variety of malignant tumours has been demonstrated over recent years. However, the role of p53 in human malignant melanoma is still unclear. Therefore, we investigated melanoma metastases from 11 patients cytogenetically and with fluorescence in situ hybridization (FISH) after short-term culture, employing a p53 region-specific probe for 17p13.1 and a probe detecting the centromere of chromosome 17. Furthermore, paraffin-embedded tissue samples from nine of these patients were investigated immunohistochemically for expression of the p53 protein. Deletions of the short arm of chromosome 17 were seen in six melanomas in cytogenetic analysis. With FISH, three malignant melanomas had clones with only one p53-allele and an additional four malignant melanomas showed a reduced number of signals at the p53 tumour-suppressor gene locus compared with signals for the centromeric region of chromosome 17. This was confirmed by immunohistochemistry. Our results suggest that the 17p11–13 region is frequently deleted in malignant melanomas and that p53 or other genes located on this band might contribute to the malignant potential of advanced melanoma.

**Keywords:** melanoma; p53; deletion; cytogenetics; fluorescence in situ hybridization; immunohistochemistry

Malignant melanoma (MM) is a common cancer among Caucasians and has shown a deplorable increase in incidence rates during recent decades. Early diagnosis – and consequently early surgical excision – has been greatly improved, leading to an average 5-year survival rate of up to 80%. Therefore, stable incident death rates of MM could be observed. However, the prognosis of metastatic MM still remains poor, with a long-term survival rate of about 20% in stage III patients (Balch et al, 1992; Johnson et al, 1995).

Cytogenetic analyses of cell lines derived from MM have been reported for more than 20 years (Chen et al, 1973). The data obtained are quite complex and not always unambiguous. However, the non-random involvement of chromosomes 1, 6, 7 and 9 has been published repeatedly (Chen et al, 1973; Koprowski et al, 1985; Heim et al, 1988; Trent at al, 1990; Balch et al, 1992). Deletions or loss of function of the p53 tumour-suppressor gene (p53-TSG) have been found in roughly 50% of all malignant tumours (Kamb et al, 1994). Mutations of the p53-TSG have been found to be characteristic for carcinogenes and biochemical mechanisms responsible for genetic lesions in DNA that cause cancer (Harris et al, 1993). Loss of function of p53 has been reported for colon, oesophageal squamous cell, pancreatic and hepatic carcinoma and other malignant tumours of the brain, lung, breast and ovary (Levine, 1993). Despite numerous studies, no clear information on the role of p53 in MM is available to date (Stretch et al, 1991; Akslen et al, 1992; Catresana et al, 1993; Piepkorn, 1994; Kanoko et al, 1995; Poremba et al, 1995; Saez-Santamaría et al, 1995; Talve et al, 1996). The majority of cultured MM cell lines is described to express p53 protein as assessed by monoclonal antibody binding. Point mutations in the p53 coding sequence (usually cytosine to thymidine) have been found in 30% of cases (Piepkorn, 1994). Stretch et al report that 73% of primary and 93% of metastatic melanomas express mutant p53 protein (Stretch et al, 1991). However, there are also reports describing a reduced p53 expression in metastatic MM compared with primary lesions (Catresana et al, 1993).

To investigate the role of p53-TSG in metastatic MM, we analysed 11 metastatic MM derived from 11 patients cytogenetically by chromosome banding techniques and FISH after short-term culture. In addition, paraffin-embedded tissues of 9 of the 11 MM were examined immunohistochemically using DO-7, an antibody reacting with both wild-type and mutant p53 protein.

*Received 26 September 1997*
*Revised 5 March 1998*
*Accepted 1 April 1998*

*Correspondence to:* C Marosi

### MATERIAL AND METHODS

### Material

Eleven surgically removed, histologically confirmed metastatic MM (ME 1 to ME 11) from 11 patients were minced with scissors and prepared for short-term culture by standard methods (Pederson et al, 1986). Patients' data are briefly described in Table 1. For preparing MM specimens for cytogenetic investigation, collagenase (Worthington Biochemical Corporation, NJ, USA) 200 U $ml^{-1}$ was used over night.

The next day, cells were washed three times to start cultivation in a collagenase-free medium with RPMI-1640 medium (Sera-lab,

**Table 1** Patients' data and clinical information of the patients as far as available

| Case | Initials | Sex | PM | Localization of PM | Date and localization of metastases | Prior treatment |
|---|---|---|---|---|---|---|
| ME 1 | DP<br>7.3 .'41 | M | 1990: PM of unknown type | Right cheek | 1991: cervical LN, right parotis<br>1992: ing and perianal LN<br>1994: multiple sc metastases<br>1994: liver, kidney, peritoneum, lung, mediastinum<br>11. 1994: right tibia | DTIC<br>carboplatin<br>DTIC/<br>muphoran |
| ME 2 | ZE<br>1.9.'46 | F | 1991: NMM<br>C IV, B 2 mm | Trunk | 12. 1993 + 9. 1994: left axillary LN<br>9. 1994: trunk, sc<br>12. 1994: trunk, sc<br>12. 1994: lung | DTIC<br>Intron A<br>IFN-α |
| ME 3 | BE<br>27.5.'34 | F | 1993: NMM<br>C IV, B 1, 96 mm | Unknown | **4. 1995**: **left ing LN** | – |
| ME 4 | PH<br>20.3.'14 | F | 1953: PM of unknown type | Right calf | **5. 1995**: **right ing LN** | – |
| ME 5 | SF<br>8.10.'20 | M | Unknown | Unknown | **8. 1995**: **trunk, sc** | – |
| ME 6 | LO<br>9.7.'12 | M | 10. 1994:<br>C III, B 2, 7 mm | Left calf | **6. 1995**: **left ing LN** | – |
| ME 7 | RF<br>23.10.'37 | M | 1989: NMM<br>C IV, B 1, 97 mm | Reg scapularis dex | **9. 1995**: **right axillary LN** | – |
| ME 8 | BK<br>2.9.'35 | M | Unknown | Unknown | **9. 1995**: **left axillary LN** | – |
| ME 9 | DA<br>9.5.'27 | M | 1993: SSM | Left ear | **10. 1995**: **LN at parotic gland** | – |
| ME 10 | NA<br>16.2.'31 | M | 1987: C IV,<br>B 1, 96 mm | Left calf | **4. 1995**: **left ing LN** | – |
| ME 11 | SF<br>5.9.'24 | M | 10. 1995: PM of unknown type | Right shoulder | **11. 1995**: **right axillary LN** | – |

Date and localization of the investigated specimens are in bold. Birthdates of the patients are presented under their initials. B, Breslow; C, Clark; DTIC, dacarbazine; IFN-α; interferon alpha; LN, lymph node; NMM, nodular malignant melanoma; PM, primary melanoma; sc, subcutaneous; SSM, superficially spreading melanoma; ing, inguinal.

Crawley-Down, Sussex, UK; JRM-Biosciences, Kansas City), 10% fetal calf serum (FCS) (Gibco BRL, Life Technologies, NY, USA), 1% AB-AM-solution (10 000 U ml$^{-1}$ Penicillin-G-Sodium, 10 000 μg ml$^{-1}$ streptomycin-sulfate, 25 μg ml$^{-1}$ amphotericin-B in 0.85% sodium chloride solution, Gibco BRL), 1% L-glutamine (Gibco BRL), 1% sodium-pyruvate (JRM Biosciences), Geneticin (Gibco BRL) 100 μg ml$^{-1}$. Cells were cultured for 2–72 h to avoid in vitro artefacts. Metaphase cells were prepared according to standard techniques using colcemid 0.5 μg ml$^{-1}$ treatment, hypotonic shock in 0.075 M potassium chloride and fixation in a 3:1 mixture of methanol and glacial acetic acid (Pederson et al, 1986; Drach, 1994).

### Karyotype analysis

The following banding techniques were used: Q-banding with quinacrine and R-banding with acridine orange for ME 1, 2, 4 and 5 after denaturation in phosphate buffer. ME 7, 8, 10 and 11 were R-C-banded using chromomycin, distamycin and DAPI (4′–6′-diamidino-2-phenylindole-2HCl) as described (Schweitzer et al, 1994). The karyotypes were classified according to ISCN 1995 (Mitelman, 1995).

### FISH

For our investigation, two probes were used simultaneously in a dual-colour FISH assay: an α-satellite DNA probe specific for the centromeric region of chromosome 17 (labelled with spectrum-green) and a probe specific for the locus 17p13.1 (labelled with spectrum-orange). Both probes were purchased from Vysis (Vysis LSI p53 DNA probe; Vysis, Stuttgart, Germany). FISH of interphase nuclei was performed as previously described (Drach et al, 1995).

For prehybridization, slides were immersed in 0.1 N hydrochloric acid/0.05% Triton-X-100, washed twice in saline sodium citrate (SSC) and ethanol (100%). DNA was denatured by incubation with formamide (70% in 2 × SSC) at 70°C for 5 min. Cells were again dehydrated using ethanol. The hybridization mixture (10 μl) was then applied to each slide. The slides were coverslipped and sealed with rubber cement. The hybridization solution contained formamide (65%; Sigma, St Louis, MO, USA), 2× SSC, dextran sulphate (10%, Oncor, Gaithersburg, MD, USA), salmon sperm DNA (100 μg ml$^{-1}$; Sigma), and the specific probes (2 μg ml$^{-1}$ final concentration). Hybridization was performed overnight at 37°C in a humidified chamber. Post-hybridization

*© Cancer Research Campaign 1999*

**Table 2** Cytogenetic analysis of eight cases of MM. ME 3, ME 6 and ME 9 were not evaluable by karyotype analysis

| Case | Results |
|---|---|
| ME 1 | 54–64, XXXY, +der(1)t(1;10)(p33;p12), +i(lq), +der(2)t(2;6)(p21;q14), +i(2q), +del(3)(q24)×2, +4, t(7;22)(p12;p11), +8, +8, +del(9)(p21), –10, +11, –12, +13, +13, +18, +21, +22, +22[cp6] |
| ME 2 | 45, XX, del(1)(p33), dup (1)(p22p33), del(9)(q33), del(12)(p12), –13, t(17;?)(p12;?), –19, –21, M(1)[cp2] |
| ME 3 | Not analysable |
| ME 4 | 46, XX [4]/43–47, XX, del(1)(p22), del(2)(p16p22), –5, del(5)(q12q14), +6, –7, del(7)(p12p21), i(8q), del(10)(p12), –11, t(15;15)(q10;q10), del(17)(p13), –19, –20, –22 [cp10] |
| ME 5 | 52–75, XXYY or del(X)(q22q25), +1, del(2)t(2;?)(q35;?), +del(3)(q21q31), –5 or del(5)(q15q23), +del(7)(p12p14)×2, +8, del(9)(p21)×2, del(10)(p13), –12, –15, del(17)(p13), +18, +19, i(20p) [cp 10] |
| ME 6 | Not analysable |
| ME 7 | 73–78, XXXY, +del(1)q32), del(2)p21), +3 or +der(3)t(3;?)(q26;?), –4×2, +t(4;5)(q13;q12)×2, +der(8)t(8;?)(p21;?)×3, del(9)(p21)×2, –10 or del(10)(p13), –11, +12, t(13;14)(q10;q10), +15, t(15;15)(q25;q13), +16, del(17)(p13) or del(17)(p11), –21, del(22)(q12) [cp 7] |
| ME 8 | 46, XY [cp 5]/46, XY, del(9)(p21) [cp 7] |
| ME 9 | Not analysable |
| ME 10 | 41–42, X, –Y, –4, –6, –9, –10, –14, hsr (4) ×2 [cp3]<br>78–82, XY, –3×2, –4, del(6)(q22), –9, del(9)(p21), –10 ×3, del(11)(q23), –12 ×3, –14, del(17)(p13), –21, hsr (4) [cp 3] |
| ME 11 | 65–78, XX or XY or XXY, dir dup (1)(p21–>pter), del(2)(p21) or del(2)(p16) or del(2)(p22), +3×2, –4, +del(6)(q15q24), +7×2, +del(8)(p21)×2, der(9)(9q22–>qter::9qter–>q22::9q22–>qter), +del(9)(p21), –11, +11p, –12, t(14;14)(q23;q21) or +t(14;15)(p10;q10), –15, del(16)(p12)×2, –17, del(17)(p11), +19, +20, +22, [cp6] |

washes consisted of three rinses in 50% formamide/2 × SSC at 45°C and two rinses in 2 × SSC at 37°C. Finally, nuclei were counterstained with DAPI. Cells were analysed under a fluorescence microscope (Olympus AH.3 Fluorescence microscope, Tokyo, Japan) equipped with a triple-bandpass filter to visualize simultaneously DAPI, spectrum-green and spectrum-orange.

Cut-off levels for the definition of aneuploidy were derived from experiments with normal human peripheral blood and bone marrow cells. Tumours exhibiting deletions or loss of the p53 allele in fewer than 10% of cells were not regarded as being deleted for the p53 gene (cut-off). At least 100 tumour cell nuclei were counted per sample.

### Immunohistochemistry

The avidin–biotin immunohistochemistry was performed by standard methods. In brief, 5-μm-thick sections of paraffin-embedded tissues of nine metastatic MM (ME 3 to 11) were cut and placed on positively charged microscope slides (Probe on Plus, Fisher Scientific Company, Pittsburgh, PA, USA). Sections were deparaffinized and antigen retrieval was performed by microwave heating in citrate buffer. Endogenous peroxidase activity was blocked with 3% hydrogen peroxide in methanol for 30 min. Before staining, sections were exposed to 2% normal goat serum. The sections were incubated with the primary antibody DO7 (Dako, Carpinteria, CA, USA; murine $IgG_{2b}$) at a dilution of 1:200 for 1 h at room temperature followed by an incubation with a biotinylated goat antimouse antibody (Dako, LSAB 2 kit) for 30 min. Peroxidase-labelled streptavidin was added for 30 min (Dako, LSAB 2 kit). The reaction product was developed using aminoethylcarbazole (Dako, AEC substrate system) and the bright red-stained immunoreactive sites could easily be distinguished from the melanin pigment. The sections were briefly counterstained with Mayer’s haematoxylin. Negative controls included species and isotype-matched antibodies (mouse $IgG_{2b}$, Dako) and positive controls with anti-S 100 antibody (Dako) were performed on all specimens.

Tumours of the human melanoma cell line 518A2 grown in severe combined immunodeficient (SCID) mice were used as additional positive control for p53 staining. This cell line has a p53 mutation and the protein can be detected in vitro and in vivo by Western blot and immunohistochemistry.

Only specimens with nuclear labelling and staining of more than 1% of the cells examined were considered to be positive for p53 (Kanoko et al, 1995). A minimum of 1000 cells per specimen was evaluated.

### RESULTS

### Karyotype analysis

The composite karyotypes of eight analysable metastatic MM (ME 1, 2, 4, 5, 7, 8, 10, 11) are shown in Table 2 and demonstrate a non-random pattern of karyotypic aberrations in advanced MM. According to our data, aberrations of chromosomes 1 and 9 were most frequently observed. Both chromosomes were involved in

© Cancer Research Campaign 1999

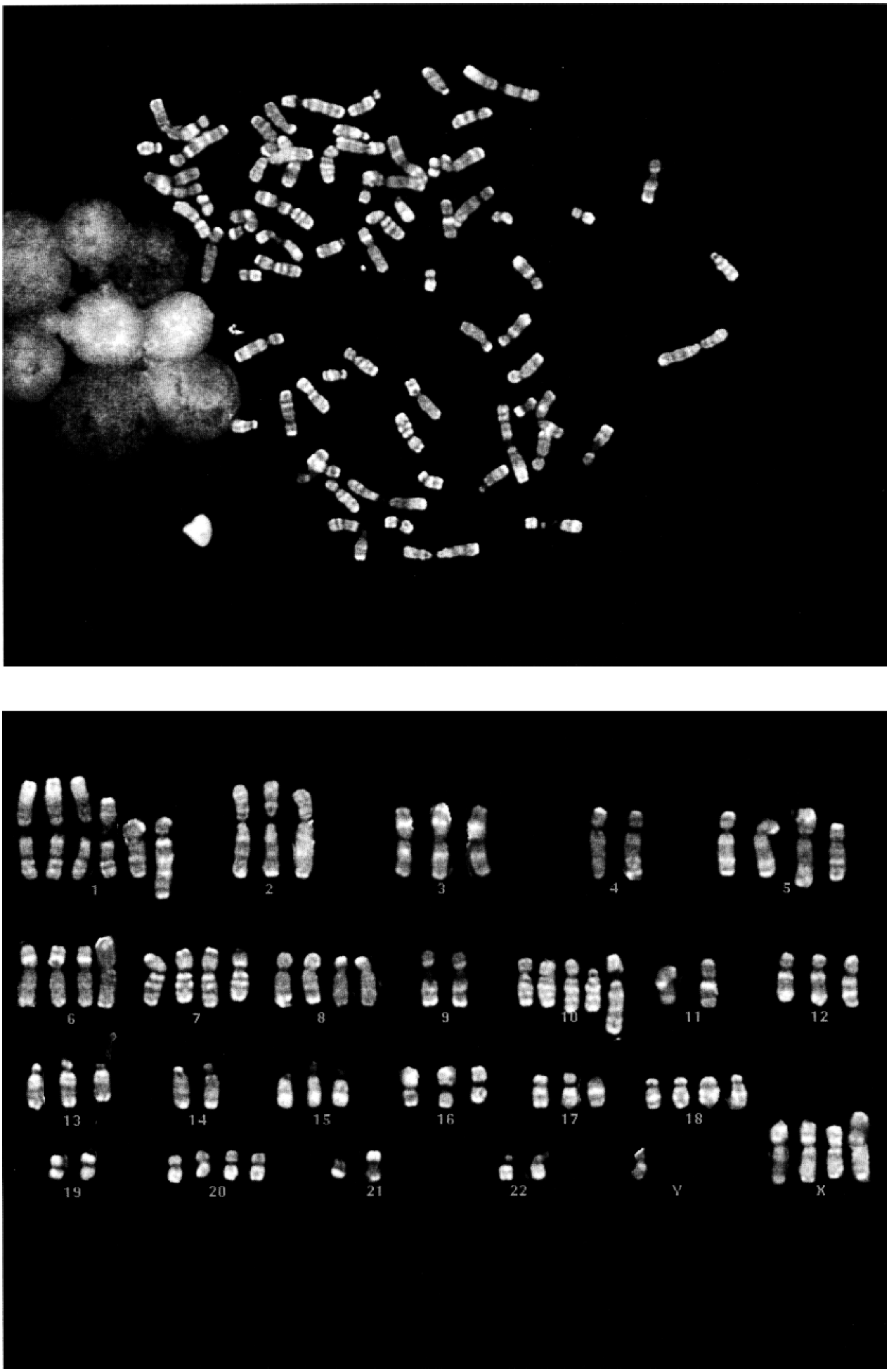

**Figure 1** A representative R-banded karyotype of ME 7 is shown. The breakpoint on chromosome 17 is in this case located on 17p11. The complete karyogram of this hypertriploid cell reads as follows: 75, XXXY, +t(X;12)(p10;q10), +del(1)(p32), +del(1)(p22), t(1;11)(q12;q25), –4, +del(5)(q11q13), der(6)t(6;?)(p12;?), +del(7)(p21), +der(8)t(8;?)(p22;?)×2, –9, +del(10)(p11), der(?10)t(?10;16)(p10;q10), t(12;18)(q21;q21), –14, del(17)(p11), +18, –19, +20, –21, t(20;21)(q12;p13), –22

 *© Cancer Research Campaign 1999*

**Table 3** FISH data shown as a percentage

| Case | % *n* (p53) = *n* (#17) | *n* (p53) = 0–1 | *n* (#17) > *n* (p53) | Cells evaluated |
|---|---|---|---|---|
| ME 1 | 88, 12 | 0 | 7, 92 | 101 |
| ME 2 | 74, 71 | 16, 82 | 24, 36 | 107 |
| ME 3 | – | – | – | 0 |
| ME 4 | 70, 59 | 0, 98 | 20, 59 | 102 |
| ME 5 | 26, 67 | 10, 48 | 73, 33 | 105 |
| ME 6 | – | – | – | 0 |
| ME 7 | 16, 83 | 26, 73 | 84, 17 | 101 |
| ME 8 | – | – | – | 0 |
| ME 9 | 64, 08 | 1, 94 | 35, 92 | 103 |
| ME 10 | 24, 75 | 26, 73 | 75, 25 | 101 |
| ME 11 | 67 | 1 | 31 | 100 |

*n*(p53), number of p53 signals, *n*(#17): number of centromeres of chromosome 17

**Table 4** Results of karyotypic analysis (metaphase cytogenetics), FISH and immunohistochemistry

| Case | KA data | FISH data Deletions (%) | FISH data Addition (%) | Immunohistochemistry |
|---|---|---|---|---|
| ME 1 | – | 7, 92 | 3, 96 | Not detected |
| ME 2 | t(17;?)(p12;?) | 24, 36 | 0, 93 | Not detected |
| ME 4 | del(17)(p13) | 20, 59 | 8, 82 | < 1% positive cells |
| ME 5 | –17[2c], del(17)(p13) | 73, 33 | 0 | < 1% positive cells |
| ME 7 | del(17)(p13) or del(17)(p11) | 84, 17 | 0 | < 1% positive cells |
| ME 8 | – | Not detectable | Not detectable | < 1% positive cells |
| ME 9 | Not detectable | 35, 92 | 0 | < 1% positive cells |
| ME 10 | del(17)(p13) | 75, 25 | 0 | < 1% positive cells |
| ME 11 | –17, del(17)(p11) | 31 | 2 | < 1% positive cells |

For karyotype analysis, only chromosomal aberrations of chromosome 17 are shown. The numbers of the column 'FISH data' are values in per cent of nuclei with deletions or additions. Melanomas that were not detectable either with karyotyping or with FISH (ME 3 and 6) are excluded from this table. –, detectable, but no aberration on chromosome 17 could be observed; [2c], in two cells.

seven of eight analysable melanomas, followed by chromosomes 8 and 17 involved in six and chromosomes 2 and 12 in five tumours each. The X chromosomes were affected in two cases. No abnormalities in Y chromosomes could be detected.

The most common change was a deletion of the band p21 on chromosome 9 in six MM, where the putative tumour-suppressor gene *MTS-1*, encoding for the cyclin-dependent kinase inhibitor 2, is located. Next in terms of frequency was the deletion of 17p13 in four MM (ME 4, 5, 7 and 10). Adding one patient with a deletion on 17p11 of ME 11 and the unbalanced translocation t(17;?)(p12;?) of ME 2 together with del(17)(p13), six melanomas showed deletions of the region 17p11–13 by using karyotype analysis (Figure 1). The breakpoints of all these deletions were proximal to the p53-TSG locus 17p13.1. Extending these findings we used FISH to confirm the frequency of the deletions of p53-TSG locus in MM.

### FISH

Eight MM (ME 1, 2, 4, 5, 7, 9, 10, 11) were analysable with FISH. Three MM (ME 2, 7 and 10) showed mono-allelic deletions of the p53-TSG locus (cells with 0–1 p53 signals). Simultaneously, a gain of chromosome 17 centromere signals (C17-s) up to eight signals was found in these cases. In tumour ME 2 16.82% of the cells were found to have no or one signal for the p53-TSG locus (p53-s), and a further 7.54% of the cells showed fewer p53 signals than C17-s (see Table 3). For ME 7 26.73% of the cells were seen to be monozygous for p53-s, whereas a further 57.44% showed less p53-TSG-s than C17-s compatible with an imbalance. Finally, 26.73% of the cells of ME 10 were found to be monozygous for the p53-TSG locus and another 48.52% of the cells showed less p53-s than C17-s.

Four other cases, namely ME 4, 5, 9 and 11, did not show mono-allelic deletions but polysomic tumour cells with imbalances leading to less p53-s than C17-s in a significant proportion of nuclei. The exact percentages of cells with imbalances of p53-s to C17-s are given in Table 3.

No MMs except case ME 6 were found to overexpress p53 protein as determined by immunohistochemistry. In case ME 6, 13% of the cells examined were positive for p53 protein but neither cytogenetic nor FISH data could be obtained.

© Cancer Research Campaign 1999

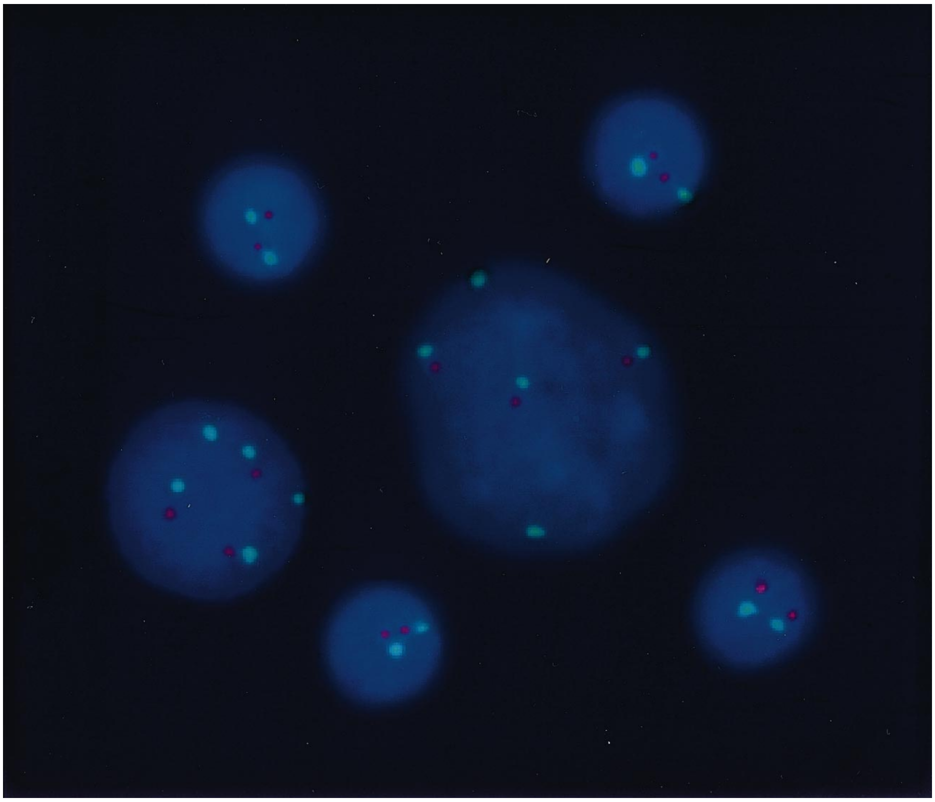

**Figure 2** FISH feature of ME 4, showing two aneuploid melanoma cells with five signals for the centromere of chromosome 17 (green signals) and three signals for the p53-TSG locus (red signals). Melanoma cells are surrounded by four lymphocytes displaying a normal hybridization pattern (two signals with both probes). Nuclei were counterstained with DAPI

### DISCUSSION

The cytogenetic and FISH data obtained in this study provide evidence that the deletion of chromosomal material of the region 17p11–13 is a frequent event in metastatic MM. According to our FISH data, three of eight analysable MM had clones with no or only one p53-TSG allele and an additional four MMs showed fewer signals of the p53-TSG locus than of the centromere of chromosome 17. Immunohistochemical investigation of nine MMs (ME 3-ME 11) showed that only one specimen overexpressed p53 protein. These observations are in concordance with our cytogenetic results, which demonstrate for the first time that loss of the p53-TSG alleles may be responsible for the reduced expression of the p53-TSG in metastatic MM and may therefore be associated with progression of this disease.

We observed good correlations between FISH and conventional cytogenetic methods. Eight of 11 MMs could be investigated with each method (karyotype analysis and FISH). Applying both methods together, information on nine MMs could be obtained. Correlation of karyotype and FISH data could be observed in seven of nine cases (see Table 4).

p53 inactivation caused by p53 point mutations has been demonstrated in haematological malignancies such as in acute myelogenous leukaemia (AML) and chronic myelogenous leukaemia (CML) blast crisis (Gaidano et al, 1993) as well as in solid tumours such as malignant gliomas (Frankel et al, 1992), medulloblastomas (Saylors et al, 1991) colon, oesophageal squamous cell, pancreatic, hepatic carcinoma and other tumours of the brain, lung, breast and ovary (Prives and Manfredi, 1993; Lee et al, 1994). In carcinomas, most of the mutations (75–80%) are missense mutations producing a faulty protein and the second allele in these cells is then lost (Carder et al, 1993; Levine, 1993). Therefore, we conclude that in the case of a deletion of a p53-TSG allele in metastatic MM, the remaining p53-TSG might be inactivated by mutations that are not detectable with immunohistochemistry, FISH or conventional cytogenetics.

As far as the expression of p53-TSG in metastatic MM is concerned, contradictory results have been reported in the past. In contrast to studies reporting that an elevated rate of p53 expression could be observed in metastatic MM (Saylors et al, 1991; Kanoko et al, 1995; Poremba et al, 1995), which may indirectly reflect p53 gene mutations, we could not confirm this phenomenon. However, we frequently detected cytogenetic loss of the 17p11–13 region. This genetic alteration may lead to loss of p53 function or decreased p53 expression, which could be confirmed by immunohistochemistry in our study.

The deletion of band p21 on chromosome 9, also found by others, appears to be the most frequent aberration in our study (Cannan-Albright et al, 1992; Fountain et al, 1992; Lynch et al, 1993; Holland et al, 1994; Skolnick et al, 1994). The deletion of 9p21, the location of *MTS-1* (*CDKN-2*, p16) and possibly further tumour-suppressor genes, was found in 57 of 99 melanoma cell lines (Cannan-Albright et al, 1992). *MTS-1* (and other genes on this region) have therefore been suspected to be factors responsible for both susceptibility and progression to MM (Saylors et al, 1991; Stretch et al, 1991).

Chromosomal and genetic deletions have been observed frequently in human tumours, and recurrently deleted gene regions

© Cancer Research Campaign 1999

have become of special interest. Our findings may shed new light on the potential significance of the tumour-suppressor gene p53 in the tumour biology of MM.

### ACKNOWLEDGEMENT

This work was supported in part by a grant from the Austrian 'Fonds zur Förderung der wissenschaftlichen Forschung' (P-10893-MED to JD).

### REFERENCES

Akslen LA and Morkve O (1992) Expression of p53 protein in cutaneous melanoma. *Int J Cancer* **52**: 13–16

Balch CM, Houghton AN, Milton GW, Sober AJ and Soong SJ (1992) *Cutaneous Melanoma*, 2nd edn. JB Lippincott: Grand Rapids, New York

Cannan-Albright LA, Goldgar DE, Meyer LJ, Lewis CM, Anderson DE, Fountain JW, Hegi ME, Wiseman RW, Petty EM, Bale AE, Olopade OI, Diaz MO, Kwiatkowsky DJ, Piepkorn MW, Zone JJ and Skolnick MH (1992) Assignment of a locus for familial melanoma, MLM, to chromosome 9p13–p22. *Science* **258**: 1148–1152

Carder P, Wyllie AH, Purdie CA, Morris RG, White S, Piris J and Bird CC (1993) Stabilised p53 facilitates aneuploid clonal divergence in colorectal cancer. *Oncogene* **8**: 1397–1401

Catresana JS, Rubio M-P and Vazquez JJ (1993) Lack of allelic deletion and point mutation as mechanism of p53 activation in human malignant melanoma. *Int J Cancer* **55**: 562–565

Chen TR and Shaw MW (1973) Stable chromosome changes in a human malignant melanoma. *Cancer Res* **33**: 2042–2047

Drach J (1994) Interphasenzytogenetik mittels Fluoreszenz-In-situ-Hybridisierung (FISH) In *Methoden der disgnostischen Hämatologie*, Huber H, Löffler H, Faber V (eds), pp. 119–124, Springer Verlag: Berlin

Drach J, Angerler J, Schuster J, Rothermund C, Thalhammer R, Haas OA, Jäger U, Fiegl M, Geissler K, Ludwig K and Huber H (1995): Interphase fluorescence in situ hydridization identifies chromosomal abnormalities in plasma cells from patients with monoclonal gammopathy of undetermined significance. *Blood* **86**: 3915–3921

Fountain JW, Karayiorgou M, Ernstoff MS, Kirkwood JM, Vlock DR, Titus-Ernstoff L, Bouchard B, Vijayasaradhi S, Houghton AN, Lahti J, Kidd VJ, Housman DE and Dracopoli NC (1992) Homozygous deletions within human chromosome band 9p21 in melanoma. *Proc Natl Acad Sci USA* **89**: 10557–10561

Frankel RH, Bayona W, Koslow M and Newcomb EW (1992) P53 mutations in human malignant gliomas: comparison of loss of heterozygosity with mutations frequency. *Cancer Res* **52**: 1427–1433

Gaidano G, Serra A, Guerrasio A, Rege-Cambrin G, Mazza U and Saglio G (1993) Genetic analysis of p53 and RB 1 tumour-suppressor-genes in blast crisis of chronic myeloid leukemia. *Ann Haematol* **68**: 3–7

Harris CC (1993) p53: at the crossroads of molecular carcinogenesis and risk assessment. *Science* **262**: 1980–1981

Heim S, Mandahl N, Arheden K, Giovanella BC, Yim SO, Stehlin Jr JS, Mitelman F (1988) Multiple karyotypic abnormalities, including structural rearrangements of 11p, in cell lines from malignant melanoma. *Cancer Genet Cytogenet* **35**: 5–20

Holland EA, Beaton SC, Edwards BG, Kefford RF and Mann GJ (1994) Loss of heterozygosity and homozygous deletions on 9p21–22 in melanoma. *Oncogene* **9**: 1361–1365

Johnson TM, Smith JW, Nelson BR and Chang A (1995) Continuing medical education: current therapy for cutaneous melanoma. *J Am Acad Dermatol* **32**(5): 689–707

Kamb A, Gruis NA, Weaver-Feldhaus J, Liu Q, Harshman K, Tavtigian SV, Stockert E, Day III RS, Johnson BE and Skolnick MH (1994) A cell cycle regulator potentially involved in genesis of many tumour types. *Science* **264**: 436–450

Kanoko M, Ueda M, Nagano T and Ichihashi M (1995) Expression of p53 protein in melanoma progression. *J Dermatol Sci* **12**: 97–103

Koprowski H, Herlyn M, Balaban G, Parmiter A, Ross A and Nowell P (1985) Expression of the receptor for epidermal growth factor correlates with increased dosage of chromosome 7 in malignant melanoma. *Somatic Cell Mol Genet* **11**: 297–302

Kuerbitz SJ, Plunkett BB, Walsh WV and Kastan MB (1992) Wild-type p53 is a cell cycle checkpoint determinant following irradiation. *Proc Natl Acad Sci USA* **89**: 7491–7495

Lee HM, Abrahamson JJA and Bernstein A (1994) DNA damage, oncogenesis and the p53 tumour-suppressor gene. *Mutation Res* **307**: 573–581

Levine AJ (1993) The tumour suppressor genes. *Annu Rev Biochem* **62**: 623–651

Lynch HT, Fusaro RM, Sandberg AA, Bixeman HA, Johnsen LR, Lynch JF, Ramesch KH and Leppert M (1993) Chromosome instability and the FAMMM syndrome. *Cancer Genet Cytogenet* **71**: 27–39

Mitelman F (1995) *An International System for Human Cytogenetic Nomenclature* Mitelman F (ed.). S. Karger: Basle

Pederson IM, Bennet JW and Wang N (1986) Nonrandom chromosome structural aberrations and oncogene loci in human malignant melanoma. *Cancer Genet Cytogenet* **20**: 11–27

Piepkorn MW (1994) Genetic basis of susceptibility to melanoma. *J Am Acad Dermatol* **31**: 1022–1039

Pietenpol JA and Vogelstein B (1993) No room at the p53 inn. Nature (London) **365**: 17–18

Poremba C, Yandell DW, Metze D, Kamanabrou D, Böcker W and Dockhorn-Dworniczak B (1995) Immunohistochemical detection of p53 in melanomas with rare p53 gene mutations is associated with mdm-2 overexpression. *Oncol Res* **7**: 331–339

Prives C and Manfredi JJ (1993) The p53 tumour suppressor protein: meeting review. *Genes Dev* **7**: 529–534

Saez-Santamaría MC, McNutt NS, Bogdany JK and Shea CR (1995) P53 expression is rare in cutaneous melanomas. *Am J Dermatopathol* **17**: 344–349

Saylors RL, Sidransky D, Friedman HS, Bigner SH, Bigner DD, Vogelstein B and Brodeur GM (1991) Infrequent p53 gene mutations in medulloblastomas. *Cancer Res* **51**: 4721–4723

Schweitzer D and Ambros PF (1994) Chromosome banding: stain combinations for specific regions. In *Methods in Molecular Biology*, Vol. 29: *Chromosome Analysis Protocols*, Gosden JR (ed), pp. 97–112. Humana Press: Totowa, NJ

Skolnick MH, Cannon-Albright LA and Kamb A (1994) Genetic predisposition to melanoma. *Eur J Cancer* 30A, **13**: 1991–1995

Stretch JR, Gatter KC, Ralfkiaer E, Lane DP and Harris AL (1991) Expression of mutant p53 in melanoma. *Cancer Res* **51**: 5976–5979

Talve L, Kainu J, Collan Y and Ekfors T (1996) Immunohistochemical expression of p53 protein, mitotic index and nuclear morphometrie in primary malignant melanoma of the skin. *Pathol Res Pract* **192**: 825–833

Trent JM, Meyskens FL, Salmon SE, Ryschon K, Leong SP, Davis JR and McGee DL (1990) Relation of cytogenetic abnormalities and clinical outcome in metastatic melanoma. *N Engl J Med* **322**: 1508–1511

© Cancer Research Campaign 1999

## References

[PDF_00385] Akslen L. A., Mørkve O. (1992). Expression of p53 protein in cutaneous melanoma.. Int J Cancer.

[PDF_00389] Cannon-Albright L. A., Goldgar D. E., Meyer L. J., Lewis C. M., Anderson D. E., Fountain J. W., Hegi M. E., Wiseman R. W., Petty E. M., Bale A. E. (1992). Assignment of a locus for familial melanoma, MLM, to chromosome 9p13-p22.. Science.

[PDF_00394] Carder P., Wyllie A. H., Purdie C. A., Morris R. G., White S., Piris J., Bird C. C. (1993). Stabilised p53 facilitates aneuploid clonal divergence in colorectal cancer.. Oncogene.

[PDF_00397] Castresana J. S., Rubio M. P., Vázquez J. J., Idoate M., Sober A. J., Seizinger B. R., Barnhill R. L. (1993). Lack of allelic deletion and point mutation as mechanisms of p53 activation in human malignant melanoma.. Int J Cancer.

[PDF_00400] Chen T. R., Shaw M. W. (1973). Stable chromosome changes in human malignant melanoma.. Cancer Res.

[PDF_00402] Drach J., Angerler J., Schuster J., Rothermundt C., Thalhammer R., Haas O. A., Jäger U., Fiegl M., Geissler K., Ludwig H. (1995). Interphase fluorescence in situ hybridization identifies chromosomal abnormalities in plasma cells from patients with monoclonal gammopathy of undetermined significance.. Blood.

[PDF_00410] Fountain J. W., Karayiorgou M., Ernstoff M. S., Kirkwood J. M., Vlock D. R., Titus-Ernstoff L., Bouchard B., Vijayasaradhi S., Houghton A. N., Lahti J. (1992). Homozygous deletions within human chromosome band 9p21 in melanoma.. Proc Natl Acad Sci U S A.

[PDF_00414] Frankel R. H., Bayona W., Koslow M., Newcomb E. W. (1992). p53 mutations in human malignant gliomas: comparison of loss of heterozygosity with mutation frequency.. Cancer Res.

[PDF_00417] Gaidano G., Serra A., Guerrasio A., Rege-Cambrin G., Mazza U., Saglio G. (1994). Genetic analysis of p53 and RB1 tumor-suppressor genes in blast crisis of chronic myeloid leukemia.. Ann Hematol.

[PDF_00420] Harris C. C. (1993). p53: at the crossroads of molecular carcinogenesis and risk assessment.. Science.

[PDF_00422] Heim S., Mandahl N., Arheden K., Giovanella B. C., Yim S. O., Stehlin J. S., Mitelman F. (1988). Multiple karyotypic abnormalities, including structural rearrangements of 11p, in cell lines from malignant melanomas.. Cancer Genet Cytogenet.

[PDF_00426] Holland E. A., Beaton S. C., Edwards B. G., Kefford R. F., Mann G. J. (1994). Loss of heterozygosity and homozygous deletions on 9p21-22 in melanoma.. Oncogene.

[PDF_00429] Johnson T. M., Smith J. W., Nelson B. R., Chang A. (1995). Current therapy for cutaneous melanoma.. J Am Acad Dermatol.

[PDF_00432] Kamb A., Gruis N. A., Weaver-Feldhaus J., Liu Q., Harshman K., Tavtigian S. V., Stockert E., Day R. S., Johnson B. E., Skolnick M. H. (1994). A cell cycle regulator potentially involved in genesis of many tumor types.. Science.

[PDF_00435] Kanoko M., Ueda M., Nagano T., Ichihashi M. (1996). Expression of p53 protein in melanoma progression.. J Dermatol Sci.

[PDF_00437] Koprowski H., Herlyn M., Balaban G., Parmiter A., Ross A., Nowell P. (1985). Expression of the receptor for epidermal growth factor correlates with increased dosage of chromosome 7 in malignant melanoma.. Somat Cell Mol Genet.

[PDF_00441] Kuerbitz S. J., Plunkett B. S., Walsh W. V., Kastan M. B. (1992). Wild-type p53 is a cell cycle checkpoint determinant following irradiation.. Proc Natl Acad Sci U S A.

[PDF_00444] Lee J. M., Abrahamson J. L., Bernstein A. (1994). DNA damage, oncogenesis and the p53 tumour-suppressor gene.. Mutat Res.

[PDF_00446] Levine A. J. (1993). The tumor suppressor genes.. Annu Rev Biochem.

[PDF_00447] Lynch H. T., Fusaro R. M., Sandberg A. A., Bixenman H. A., Johnsen L. R., Lynch J. F., Ramesh K. H., Leppert M. (1993). Chromosome instability and the FAMMM syndrome.. Cancer Genet Cytogenet.

[PDF_00452] Pedersen M. I., Bennett J. W., Wang N. (1986). Nonrandom chromosome structural aberrations and oncogene loci in human malignant melanoma.. Cancer Genet Cytogenet.

[PDF_00455] Piepkorn M. W. (1994). Genetic basis of susceptibility to melanoma.. J Am Acad Dermatol.

[PDF_00457] Pietenpol J. A., Vogelstein B. (1993). Tumour suppressor genes. No room at the p53 inn.. Nature.

[PDF_00460] Poremba C., Yandell D. W., Metze D., Kamanabrou D., Böcker W., Dockhorn-Dworniczak B. (1995). Immunohistochemical detection of p53 in melanomas with rare p53 gene mutations is associated with mdm-2 overexpression.. Oncol Res.

[PDF_00463] Prives C., Manfredi J. J. (1993). The p53 tumor suppressor protein: meeting review.. Genes Dev.

[PDF_00465] Saenz-Santamaría M. C., McNutt N. S., Bogdany J. K., Shea C. R. (1995). p53 expression is rare in cutaneous melanomas.. Am J Dermatopathol.

[PDF_00467] Saylors R. L., Sidransky D., Friedman H. S., Bigner S. H., Bigner D. D., Vogelstein B., Brodeur G. M. (1991). Infrequent p53 gene mutations in medulloblastomas.. Cancer Res.

[PDF_00470] Schweizer D., Ambros P. F. (1994). Chromosome banding. Stain combinations for specific regions.. Methods Mol Biol.

[PDF_00473] Skolnick M. H., Cannon-Albright L. A., Kamb A. (1994). Genetic predisposition to melanoma.. Eur J Cancer.

[PDF_00475] Stretch J. R., Gatter K. C., Ralfkiaer E., Lane D. P., Harris A. L. (1991). Expression of mutant p53 in melanoma.. Cancer Res.

[PDF_00477] Talve L., Kainu J., Collan Y., Ekfors T. (1996). Immunohistochemical expression of p53 protein, mitotic index and nuclear morphometry in primary malignant melanoma of the skin.. Pathol Res Pract.

[PDF_00480] Trent J. M., Meyskens F. L., Salmon S. E., Ryschon K., Leong S. P., Davis J. R., McGee D. L. (1990). Relation of cytogenetic abnormalities and clinical outcome in metastatic melanoma.. N Engl J Med.

